# Case Report: PsAPSASH syndrome: an alternative phenotype of syndromic hidradenitis suppurativa treated with the IL-17A inhibitor secukinumab

**DOI:** 10.12688/f1000research.52100.1

**Published:** 2021-05-13

**Authors:** Georgios Nikolakis, Katja Kreibich, Aristeidis Vaiopoulos, Katarzyna Kaleta, Joud Talas, Markus Becker, Christos C. Zouboulis

**Affiliations:** 1 Departments of Dermatology, Venereology, Allergology and Immunology, Dessau Medical Center, Brandenburg Medical School Theodor Fontane and Faculty of Health Sciences Brandenburg, Dessau, 06847, Germany; 2 European Hidradenitis Suppurativa Foundation e.V., Dessau, 06847, Germany; 3 Department of Dermatology, Jagiellonian University Medical College, Krakow, 31-008, Poland

**Keywords:** PASH, PAPASH, PASS, "hidradenitis suppurativa", SAPHO, PAPA, "acne inversa", hidradenitis, secukinumab, syndrome, arthritis, autoinflammatory, pustulosis

## Abstract

Syndromic hidradenitis suppurativa (HS) is a form of symptom constellations, which differs from the familial and genetic form and comprises predominantly osteoarticular manifestations. Many forms include pyoderma gangrenosum and acne (PASH), pyogenic arthritis (PAPASH), spondyloarthritis (PASS) and psoriatic arthritis (PsaPASH) and are categorized in the autoinflammatory syndromes. anti-TNF-α and anti-IL-1a blockade are between the therapeutic approaches that improve skin symptoms and prevent permanent osteoarticular damage. This case report refers to the successful treatment of a mixed phenotype of the aforementioned symptoms using the IL-17A inhibitor secukinumab after initial treatment with adalimumab. The therapy improved both cutaneous and reported osteoarticular symptoms. Different approaches for these recalcitrant HS syndromes are essential in order to achieve long-term remission for those patients.

## Introduction

Hidradenitis suppurativa (HS) is a chronic, debilitating inflammatory skin disorder of the terminal hair follicle characterized by the presence of nodules, abscesses, tunnels and extensive scarring in the apocrine gland-rich areas of the body. ^1^ Immune dysregulation has been implicated in HS, with a wide range of cytokines identified. Significant increase of proinflammatory cytokines IL-1β, TNF-α, IL-17 and the antiinflammatory cytokine IL-10 has been detected in lesional and perilesional skin. ^2, 3^ Apart from sporadic cases, there is a genetic background for certain HS patients, correlating with mutations in the γ-secretase genes nicastrin, presenilin enhancer 2 and presenilin. ^4^ HS has been described in association with several clinical syndromes that include comorbid disorders, such as pyogenic arthritis (PA), pyoderma gangrenosum (PG), acne, ulcerative colitis (UC) and psoriatic arthritis. Such syndromes include the triad of PG, acne and HS (PASH) alone or in combination with PA (PAPASH) or psoriatic arthritis (PsAPASH). In addition, HS can also be a feature of other syndromes such as the SAPHO syndrome, ^5^ which appear recalcitrant to treatment, even after the use of various biologics, such as anti-TNF and anti-IL-1. Here we describe a patient with a novel phenotypic variant of the syndromes described, who responded to treatment with the IL-17A inhibitor secukinumab.

**Figure 1.**
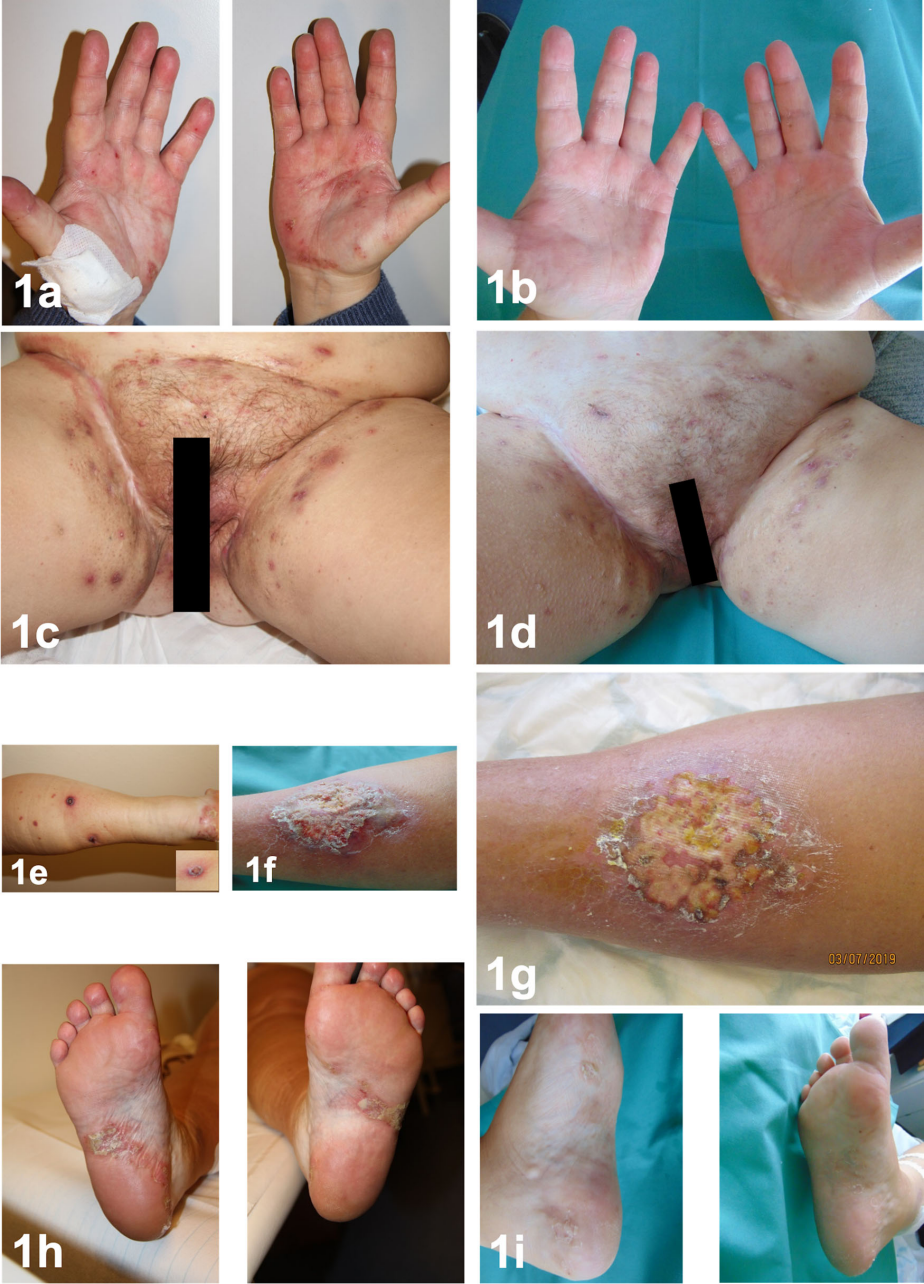
palmar psoriasis before (1a) and after (1b) treatment with secukinumab over 4 months. Manifestations of HS localized in the inguinofemoral region before (1c) and after (1d) treatment. Pyoderma gangrenosum two months after adalimumab discontinuation (1e), clinical image 4 months after secukinumab treatment (1f) and post-prednisolone i.v pulse therapy (1g). Plantar pustulosis before (1h) and after secukinumab treatment (1i).

**Figure 2.**
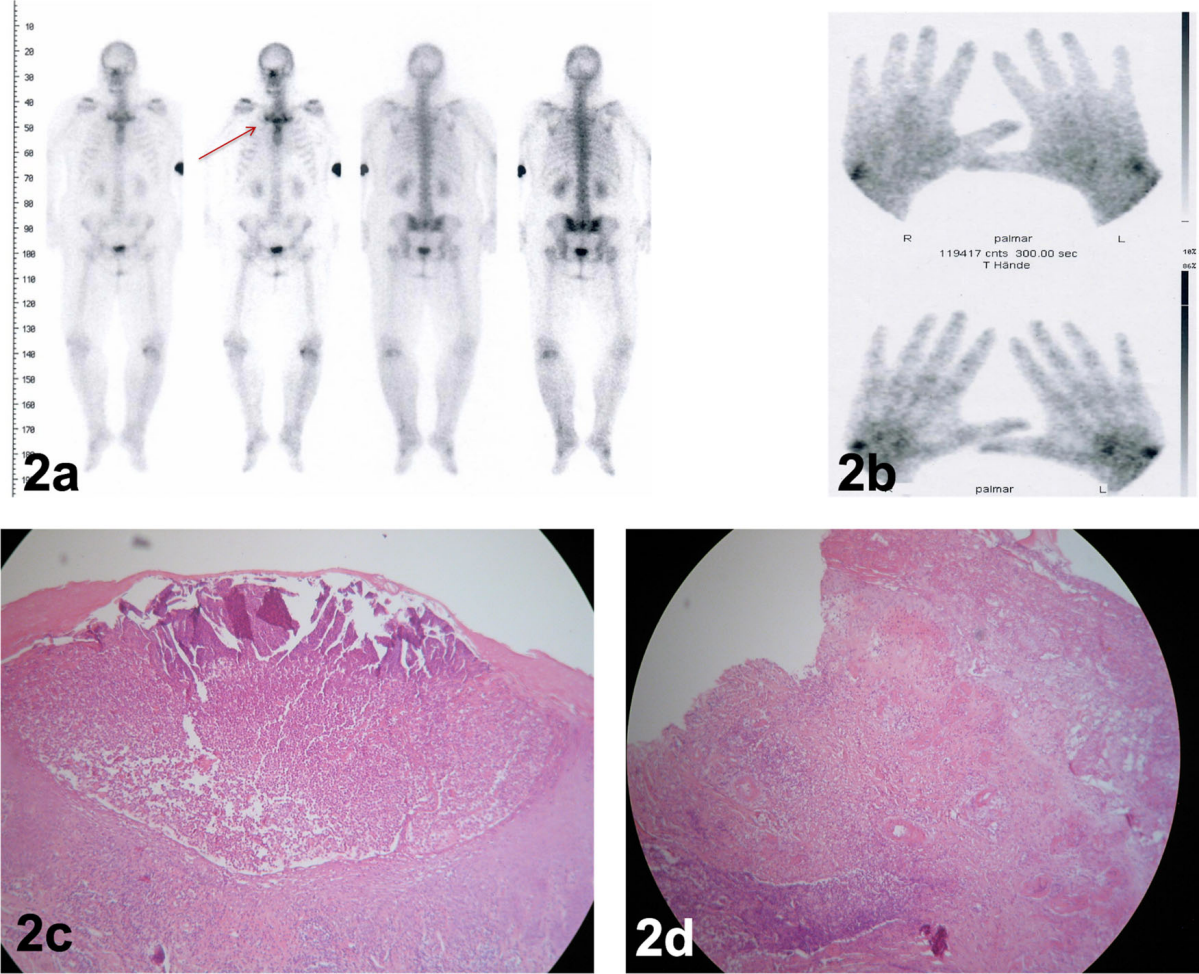
Bone scan revealing a “bull’s head” sign-like radiopharmaceutical accumulation of the costoclavicular region (2a, red arrow) and of the wrist joints (2b). (2c): histopathologic evaluation of palmar pustular eruptions revealed characteristic microabscesses of subcorneal neutrophilic accumulation. (2d): Histology of the lower-leg ulcerations demonstrated diffuse neutrophilic inflammation as a hallmark of pyoderma gangrenosum.

## Case

A 50-year-old female, Caucasian, unemployed patient was admitted to our departments suffering from HS. The patient reported a disease onset of 25 years, describing exacerbations with relapsing nodules, abscesses, and draining sinus tracts. Among the risk factors correlated with the disease, the patient was obese (BMI = 36) and a smoker (34-pack years). Moreover, she had a positive familial history, with her maternal grandmother having had severe refractory HS. Sequencing of the γ-secretase gene complex did not reveal any relevant mutations. The patient reported to having up to 10-15 stools daily. A colonoscopy was performed three years ago, which excluded inflammatory bowel disease. Twelve years after being diagnosed with HS, the patient developed acne conglobata (AC), which was treated without systemic therapy. In addition, she underwent numerous incisions and radical excisions, antibiotic treatment with doxycycline, and the combination of clindamycin and rifampicin over 3 months, according to the HS treatment guidelines, ^1, 2^ without sustained remission of the lesions. A previous 1-year therapy with isotretinoin did not improve the HS lesions. The patient was included in a clinical trial combining weekly administration of 40 mg adalimumab s.c. vs. placebo for 3 months, followed by continuation of adalimumab treatment in the same dose over 15 months. After this period, the patient was lost to follow-up. She described an improvement of the HS lesions and reduction of flares under adalimumab. During this time, her general practitioner discontinued adalimumab treatment, judging that the treatment lacked efficacy.

One month after discontinuation of adalimumab, the patient developed confluent erythematous pustules on the palms ( Figure 1a) and soles ( Figure 1h) with psoriasis-like scaling of the lesions followed by intermittent shoulders and knee pain and swelling. We performed a bone scan, which showed intense radiopharmaceutical accumulation of the left knee ( Figure 2a) and the wrist joints showed signs of arthritis and synovitis ( Figure 2b), with no typical pattern of psoriatic arthritis. The pattern of the bull’s head sign ( Figure 2a, red arrow) was detected. The patient did not admit having a pain or recurrent swelling of the costoclavicular region. No typical signs of osteitis were detected. Dermatohistology of the palmoplantar lesions revealed characteristic neutrophilic abscesses, compatible with pustular psoriasis ( Figure 2c).

Two months after therapy discontinuation, single, disseminated, painful pustules appeared on both thighs and lower legs ( Figure 1e), which progressed to painful ulcers ( Figure 1f) with elevated violaceous margins. The histopathological evaluation confirmed a PG ( Figure 2d). Based on the disease pathophysiology, we initiated therapy with secukinumab 300 mg s.c weekly for the first month and then monthly thereafter. Four months later, the patient demonstrated a significant remission of her HS lesions ( Figure 1d) (ΔIHS4 9 and ΔDLQI 11), joint pains and pustular psoriasis ( Figure 1b, Figure 1i), with only moderate improvement of her PG lesions. An epithelization was not observed. The PG showed no improvement and 100 mg prednisolone daily i.v. over three days was added to the treatment, which was subsequently tapered over one month. The treatment was followed by PG improvement ( Figure 1g).

## Discussion

HS can also be a main or secondary element of certain syndromes highlighted through their unique phenotypes, ^5^ known as autoinflammatory diseases (AID). AID manifests with recurrent sterile inflammation, while high autoantibody serum levels or antigen-specific lymphocytes are lacking. ^6– 9^ These syndromes combine dermatological manifestations (HS, PPP, AC, PG), musculoskeletal disorders (arthritis, synovitis, hyperostosis, osteitis) and gastroenterological manifestations (ulcerative colitis, M. Crohn). Combinations lead to already described syndromes such as PASH, ^10, 11^ PASS, ^8^ PAPASH, ^10^ PsAPASH ^12^ and SAPHO. ^13^ These disorders are characterized by aberrant release of IL-1β, which mediates the increase of tumor necrosis factor α (TNF-α), interferon γ (IFN-γ) and other chemokines, which are responsible for neutrophilic recruitment and might promote an anti-apoptotic microenvironment. ^14– 17^ IL-17 also promotes neutrophilic recruitment and activation and has a synergistic effect with TNF-α. ^18^ An imbalance of the Th17/Treg lymphocyte ratio is believed to aggravate autoinflammation and was reported both in PG and HS independently. ^19, 20^

We describe a new syndromic HS-related phenotype (pustular **Ps**oriasis, **A**rthritis, **P**G, **S**ynovitis, **A**cne, **S**uppurative **H**idradenitis). The pathophysiology based on the dysregulation of IL-17 production provided the rationale for the treatment with the IL-17A inhibitor secukinumab. Moreover, this case underlines the role of the dermatologist in diagnosing such symptoms and leading a multidisciplinary approach for these patients. Dermatological manifestations can precede the osteoarticular or other organ symptoms and timely treatment initiation might avoid irreversible complications.

## Data availability

All data underlying the results are available as part of the article and no additional source data are required.

## Consent

The patient has provided written consent for the use of all photos provided in the manuscript. The consent included potential use of the material in lecture(s) and/or publication(s).
