# Supplementary material for: Case Report: PsAPSASH syndrome: an alternative phenotype of syndromic hidradenitis suppurativa treated with the IL-17A inhibitor secukinumab
Source: F1000Res. 2021 Jul 16;10:381. Originally published 2021 May 13. [Version 2] doi: 10.12688/f1000research.52100.2 (PMC8424462; doi:10.12688/f1000research.52100.2)
Supplement: Peer review history

# Peer review: Reviewer response for version 2

**DOI:** 10.5256/f1000research.57760.r89808

**Published:** 2021-09-07

**Authors:** Mathilde Daxhelet

**Affiliations:** 1 Department of Dermatology, Erasme Hospital, Université Libre de Bruxelles, Brussels, Belgium

I approve this article with no reservation.

Are enough details provided of any physical examination and diagnostic tests, treatment given and outcomes?

Partly

Is the case presented with sufficient detail to be useful for other practitioners?

Partly

Is sufficient discussion included of the importance of the findings and their relevance to future understanding of disease processes, diagnosis or treatment?

Partly

Is the background of the case’s history and progression described in sufficient detail?

Partly

Reviewer Expertise:

Hidradenitis Suppurativa

I confirm that I have read this submission and believe that I have an appropriate level of expertise to confirm that it is of an acceptable scientific standard.

# Peer review: Reviewer response for version 2

**DOI:** 10.5256/f1000research.57760.r89809

**Published:** 2021-07-20

**Authors:** Simone Garcovich

**Affiliations:** 1 Department of Dermatology, Università Cattolica del Sacro Cuore, Rome, Italy

The authors have addressed all of the reviewers' comments and substantially improved the paper.

Are enough details provided of any physical examination and diagnostic tests, treatment given and outcomes?

Yes

Is the case presented with sufficient detail to be useful for other practitioners?

Yes

Is sufficient discussion included of the importance of the findings and their relevance to future understanding of disease processes, diagnosis or treatment?

Partly

Is the background of the case’s history and progression described in sufficient detail?

Partly

Reviewer Expertise:

Hidradenitis suppurativa

I confirm that I have read this submission and believe that I have an appropriate level of expertise to confirm that it is of an acceptable scientific standard.

# Peer review: Reviewer response for version 1

**DOI:** 10.5256/f1000research.55330.r85308

**Published:** 2021-06-07

**Authors:** Simone Garcovich

**Affiliations:** 1 Department of Dermatology, Università Cattolica del Sacro Cuore, Rome, Italy

This is a very interesting report on a exemplary case of syndromic hidradenitis suppurativa, requiring a complex systemic treatment. While potentially interesting, there are some minor points to be addressed by the authors, to further improve an already well-written case report:

 1. Regarding joint manifestations: it seems there is some minor uptake of radiotracer localized to the sacroiliac joints. Osteoarticular involvement strongly reminds the SAPHO pattern. Did the patient refer any back pain? Differential diagnosis with SAPHO should be discussed by the authors. 

 2. The authors propose a novel acronym for the reported case. In my opinion, the conclusion section should be more conservative, as there is no experimental (genetic, molecular, cytokine data etc.) supporting this concept. Furthermore, this is a heavily pre-treated patient with a long disease history of HS. Could be there be an association between the previous exposure to multiple drugs (retinoids, anti-TNFs, etc.) and the development of "paradoxical" autoinflammatory extracutaneous involvement (such as recalcitrant PG)? This could be a more interesting take on the case? Please include some additional references.

 3. Treatment: the authors should better describe the take-home messages for the therapeutic management of such complex/severe disease phenotypes. Maybe, a combination treatment strategy for complex autoinflammatory disease phenotypes is more effective than monotherapy (i.e. secukinumab alone)?

 Please include some additional references on this such as: 

- Garcovich *et al.* (2017) ^1^.
- Jin *et al.* (2019) ^2^.
- Tan *et al.* (2021) ^3^.

Are enough details provided of any physical examination and diagnostic tests, treatment given and outcomes?

Yes

Is the case presented with sufficient detail to be useful for other practitioners?

Yes

Is sufficient discussion included of the importance of the findings and their relevance to future understanding of disease processes, diagnosis or treatment?

Partly

Is the background of the case’s history and progression described in sufficient detail?

Partly

Reviewer Expertise:

Hidradenitis suppurativa

I confirm that I have read this submission and believe that I have an appropriate level of expertise to confirm that it is of an acceptable scientific standard.

## References

[1] : Drug management of neutrophilic dermatoses.*Expert Rev Clin Pharmacol*.2017;10(10) : 10.1080/17512433.2017.13567191119-112810.1080/17512433.2017.135671928715916

[2] : Pyoderma gangrenosum triggered by switching from adalimumab to secukinumab.*J Dermatol*.2019;46(3) : 10.1111/1346-8138.14611e108-e10910.1111/1346-8138.1461130192400

[3] : Pyoderma gangrenosum induced by biosimilar adalimumab in a seropositive rheumatoid arthritis patient - a paradoxical effect of adalimumab?. *Rheumatology (Oxford)*.2021; 10.1093/rheumatology/keab19410.1093/rheumatology/keab19433836074

# Response

**Published:** 2021-06-11

**Authors:** Nikolakis Georgios

We would like to thank Dr. Garcovich for approving our manuscript, their careful evaluation of the case and their contribution to its improvement.

 The important differential diagnosis of the case being a variant of SAPHO syndrome including HS and a paradoxical reaction to the switch of the biologic(s) or to the previous adalimumab treatment were also addressed. An extensive opinion on combination of treatments (IL17/TNFα) or IL-1β was included but because of the lack of controlled studies - as Dr Garcovich mentioned in their article - it cannot have a strong recommendation level.

# Peer review: Reviewer response for version 1

**DOI:** 10.5256/f1000research.55330.r85309

**Published:** 2021-05-26

**Authors:** Mathilde Daxhelet

**Affiliations:** 1 Department of Dermatology, Erasme Hospital, Université Libre de Bruxelles, Brussels, Belgium

Thank you for giving me the opportunity to review this really interesting case.

 This case report describes an mixed syndrome encountered in a patient initially suffering from HS and treated with secukinumab.

 The clinical, scintigraphic and histopathological images provided are of high quality.

 However I would like to add some details to the case description:

- The locations of the HS lesions on the patient's skin.
- Why is she unemployed? Because of HS?
- I would also add the initial severity assessment scores (IHS4 and DLQI) before secukinumab to better understand the improvement of symptoms with treatment.
- I would specify that the bull's head sign is typical of SAPHO syndrome to facilitate understanding of the case as not all doctors are experts in interpreting bone scans.

Finally, I would add to the discussion that the initial response to secukinumab is promising but long-term follow-up of the patient is needed to assess long-term remission.

Are enough details provided of any physical examination and diagnostic tests, treatment given and outcomes?

Partly

Is the case presented with sufficient detail to be useful for other practitioners?

Partly

Is sufficient discussion included of the importance of the findings and their relevance to future understanding of disease processes, diagnosis or treatment?

Partly

Is the background of the case’s history and progression described in sufficient detail?

Partly

Reviewer Expertise:

Hidradenitis Suppurativa

I confirm that I have read this submission and believe that I have an appropriate level of expertise to confirm that it is of an acceptable scientific standard, however I have significant reservations, as outlined above.

# Response

**Published:** 2021-06-11

**Authors:** Nikolakis Georgios

We would like to thank Dr. Daxhelet for their comments and their valuable contribution to improve the manuscript

 We have mentioned the initial HS scores. Unemployment was not correlated with HS. The localization of the lesions was highlighted. We added the comment about the bull's head sign and its correlation with SAPHO syndrome. A sentence was added in the discussion, underlining the importance of long-term follow-up also in combination with the other reviewer's remarks
